# A histological description of alpaca (*Vicugna pacos*) cheek teeth: Findings and anatomical variations in macroscopically normal molars

**DOI:** 10.3389/fvets.2022.972973

**Published:** 2022-10-31

**Authors:** Kirsten Proost, Carsten Staszyk, Matthieu N. Boone, Jörg Vogelsberg, Ivàn Josipovic, Lieven Vlaminck, Koen Chiers

**Affiliations:** ^1^Department of Large Animal Surgery, Anesthesia and Orthopaedics, Faculty of Veterinary Medicine, Ghent University, Merelbeke, Belgium; ^2^Institute of Veterinary-Anatomy, -Histology and -Embryology, Faculty of Veterinary Medicine, Justus-Liebig-University Giessen, Giessen, Germany; ^3^Department of Physics and Astronomy – Radiation Physics, Faculty of Science, Radiation Physics Research Group – Centre for X-ray Tomography of the UGent, Ghent University, Ghent, Belgium; ^4^Department of Pathobiology, Pharmacology and Zoological Medicine, Faculty of Veterinary Medicine, Ghent University, Merelbeke, Belgium

**Keywords:** light microscopy (LM), micro-computed tomography, alpaca (*Vicugna pacos*), histology, cheek teeth, vasodentin

## Abstract

Scientific literature on veterinary dentistry in alpacas has historically focused on the description of tooth root abscesses. However, recent studies have shown a variety of other, sometimes preceding dental conditions to be widespread in this species. To allow the development and finetuning of treatment strategies in this species, a more thorough understanding of the underlying etiopathogenesis of dental disease is required. Histological studies focusing on normal dental and surrounding tissues might serve as a basis for this purpose. Nine teeth, extracted from seven alpacas were collected. All samples were retrieved from animals that died or were euthanized for non-dental reasons. Histological sections were prepared at three different levels in each tooth and examined using light microscopy focusing on the assessment of pulp tissue, dentin, cementum, periodontal tissues and the apical region. The histological appearance of the investigated dental tissues in alpacas showed great similarities with other hypsodont species. However, a rather rare type of dentin called “vasodentin” could be identified in all examined cheek teeth. Another species-specific finding was the extremely close proximity of varying neighboring tooth roots that seemed to be responsible for inducing massive resorptive lesions. The results of this study might contribute to a better understanding of the etiopathogenesis of some dental diseases in the alpaca.

## Introduction

Dental disease in alpacas is recognized as a common and often severe health problem (1, 2). A recent field study focusing on the domesticated alpaca population residing in the Northern part of Belgium and the Southern part of the Netherlands has shown a high prevalence of dental disorders. Additionally, scientific literature reports on New World Camelids, including alpacas, have shown a significantly higher prevalence of apical infections in comparison to other herbivorous species, emphasizing the need for the further development of specialized dental care (1, 3). However, in-depth knowledge of the alpaca dentition remains limited thereby hampering a thorough understanding of the etiopathogenesis of dental disease in this species. In other hypsodont species, including different equids, studies of the normal dental anatomy have led to a better comprehension and ability to evaluate dental disorders at a gross and histological level. This has led to novel insights into diagnosis and treatment strategies of dental pathology in horses (4–6). An extensive μ-CT study of normal mandibular and maxillary cheek teeth anatomy in alpacas illustrated the presence of a complex and dynamic dental pulp architecture characterized by the age-related development of several separated pulp segments from an originally interconnected pulp system with a common pulp chamber as the central entity (7, 8). Imaging studies have also shown close contacts between roots of adjacent cheek teeth which raised questions concerning its contribution to the development and spread of infectious dental disease (7–9). Further histopathological investigations and the characterization of dental diseases in alpacas are hampered by a lack of data describing the normal microanatomical appearance of dental substances as well as pulp and periodontal tissues in alpaca cheek teeth. Therefore, the authors aimed to elucidate the structural features of healthy alpaca cheek teeth and their periodontal environment using light microscopy and μ-CT investigations in order to provide a basis to differentiate anatomical variations from pathological changes.

## Materials and methods

### Tooth selection

The study population included a total of nine normal cheek teeth originating from seven alpacas (*Vicugna pacos*) with a mean age of 6 years and 5 months ± 3 years and 3 months (range: 3–11 years) approximately. The sample pool included two maxillary first molars, two maxillary second molars, two mandibular first molars and three mandibular second molars with a dental age ranging from 2 years and 4 months to 10 years and 4 months (mean 5 years and 2 months ± 3 years and 3 months). All included animals died or were euthanized for non-dental reasons, unrelated to this study. Dental quadrants were extracted from the head using an oscillating saw and subsequently fixed in 10% buffered formalin (pH 7.4) at room temperature. Dental arches were macroscopically screened for pathological changes.

### μ-CT scanning

Selected normal dental arches without the presence of dental abnormalities were then scanned using micro-computed tomography to confirm the macroscopical screening and exclude any dental pathology, e.g apical infections and severe periodontal disease, pronounced wear abnormalities, or tooth fractures. The high resolution μ-CT scanning of eight out of nine complete mandibular and maxillary arches was performed in the custom-built scanner system HECTOR at the Ghent University Center for X-ray Tomography (10). For each sample, 2001 projection images with an exposure time of 1s per projection were acquired over an angular range of 360°. Using geometrical magnification, an isotropic voxel size in the reconstruction of 55^3^ μm^3^ was achieved. The tube was set to a high voltage of 180kV and a target (output) power of 40W, resulting in a negligible influence of the focal spot size on the image resolution. To reduce beam hardening effects, a filter of 3 mm Aluminum was used. The projection data was reconstructed into a 3D volume using the implementation of the FDK algorithm in the in-house developed package Octopus Reconstruction. A commercial 3D-rendering software package (VGStudioMAX and myVGL, Volume Graphics GmbH, Germany) was used to generate 3D renderings and orthogonal slicing of included elements and to perform specific measurements for each included cheek tooth element. Acquired two dimensional μ-CT images are used throughout the manuscript to allow clarification and visualization of the presented histological images.

### Histological processing

After μ-CT scanning, a diamond saw (Proxxon Type MBS 240/E No 27 172, Föhren, Germany) was used to extract selected elements along with their associated periodontal tissues and to cut 5 mm thick horizontal sections from the sub-occlusal region, the mid-tooth region, and the apical region of each tooth. In this way, individual samples were decalcified using a buffered ethylene diamine tetra-acetate solution (EDTA, pH 8.0) for about 8 weeks placed on a shaker table at room temperature. The decalcifying solution was changed twice a week. After decalcification, specimens were embedded in paraffin wax, 7 μm thin sections were cut and stained with Toluidin blue. Acquired samples were studied via light and differential interference contrast microscopy (Leica DM2500, Leica Microsystems GmbH, Wetzlar, Germany). Microscopic photographs were taken using a microscope camera (Leica DFC 320, Leica Microsystems GmbH, Wetzlar, Germany).

### Histological assessment

The microscopical characteristics of the dental hard substances and dental and periodontal tissues were described and alpaca specific structures were identified. Referencing to specific pulp horns and root canals was based on a specific numbering system for alpaca cheek teeth (7, 8). A classification of different types of dentin was performed based on the classification system as proposed by Dacre et al. (11) in other hypsodont species. The thickness of the cementum layer was measured at a representative thickest point. The following morphologic and morphometric details were recorded. Different types of peripheral cementum were characterized based on the number of cementoblast lacunae/100 μm^2^, the distribution pattern of cementoblast lacunae and incremental lines. The number of cementoblast lacunae/100 μm^2^ was determined based on the lacunae count in five locations of 10.000 μm^2^. The peripheral cementum layer thickness was assessed as an indicator for pressure/tension sides at the dental roots. The presence of blood vessels within the cementum was recorded. The infundibular cementum was assessed in analogy to the peripheral cementum. At the level of the periodontal ligament, thickness and direction of collagen fiber bundles was noted as well as presence and distribution of cell rest of Malassez. The endodontium was assessed by description of the pulp tissue, alignment of odontoblasts, presence of predentin and mode of calcification and alignment of dentinal tubules. In all sections the possible presence and distribution of leukocytic infiltrations were recorded. Specific findings in each histological section were visualized and compared with the previously acquired μ-CT images.

## Results

**Figure 1:**
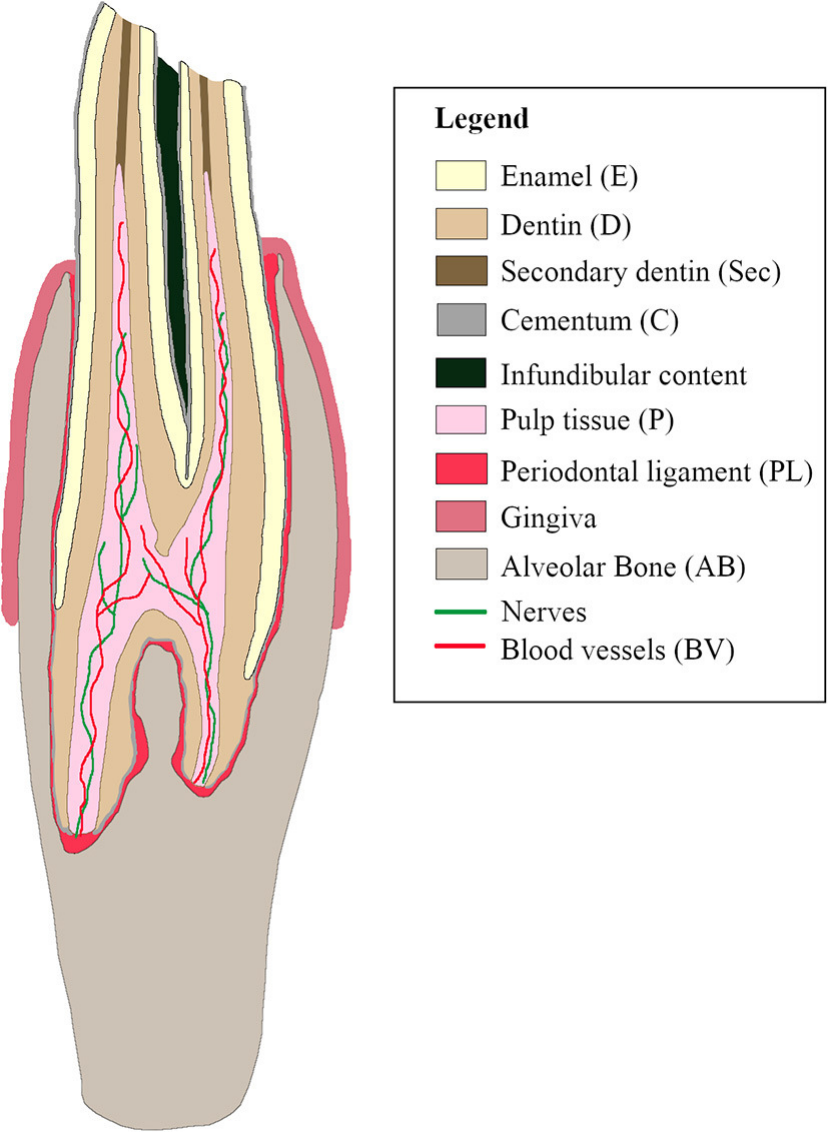
Schematic sagittal representation of a normal cheek tooth in the alpaca.

The occlusal surface of all examined cheek teeth did not show any pitting, abnormal food entrapment or specific abnormal roughness. Cheek teeth in alpacas have typical large occlusal enamel invaginations (infundibula) which can be found in a hypsodont dentition. Food entrapment at the level of these infundibula of included cheek teeth was considered normal. A schematic sagittal representation of the normal structure of a cheek tooth in the alpaca is shown in Figure 1. Given all samples were decalcified, enamel was lost due to its high mineral content, thereby impossible to evaluate histologically. A thorough microscopical evaluation of the present dentin, cementum and pulp tissue was however possible through histology. μ-CT images of regions corresponding to the histological sections allowed a comparative analysis of dentin, cementum, and pulp tissue and allowed a structural analysis of the present enamel. The general histological appearance of most structures corresponds to what has already been described in other herbivorous species including the horse, donkey and mule deer (11–13).

### Pulp tissue

**Figure 2:**
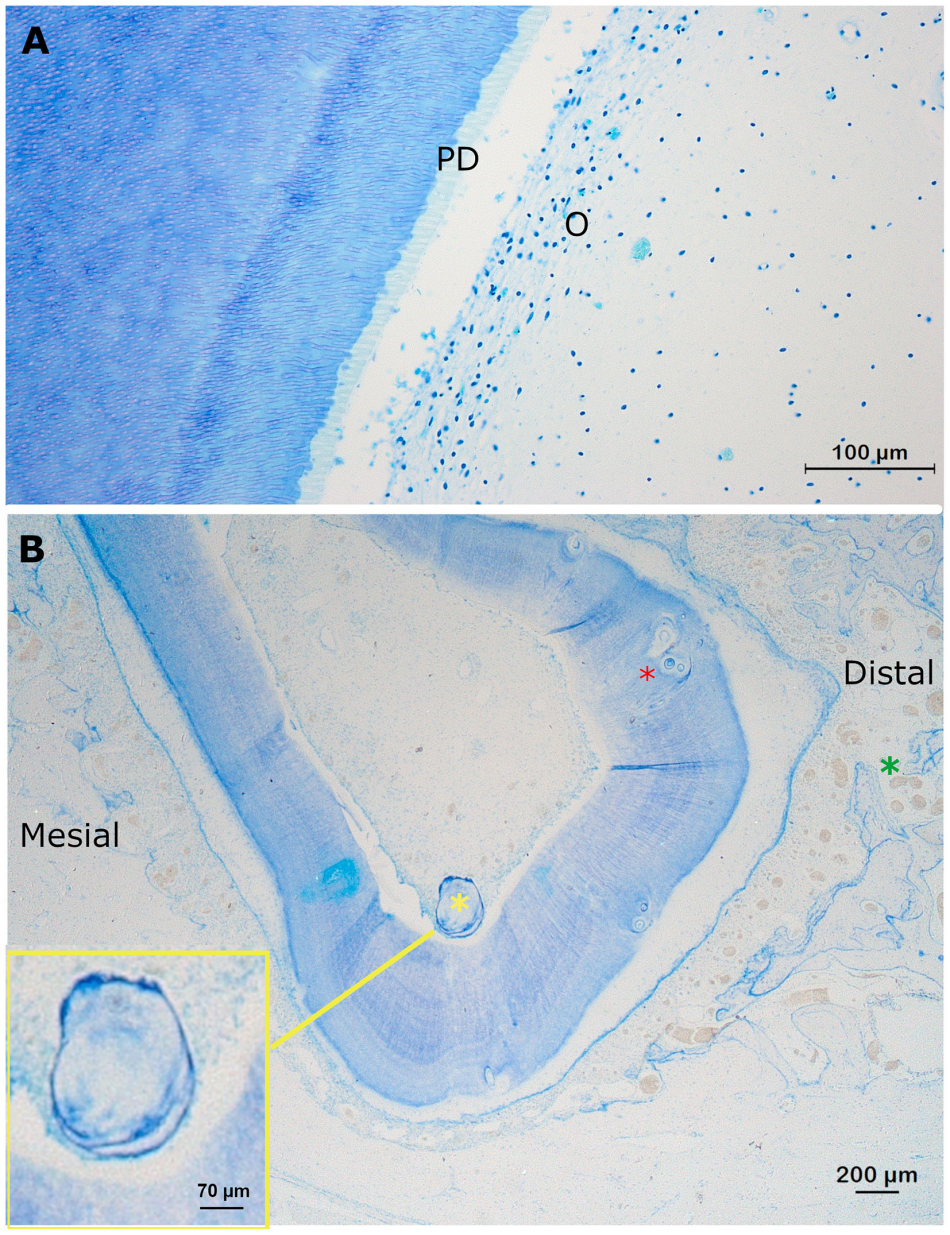
Normal findings at the level of the pulp. **(A)** Transverse section at a mid-tooth level (10 mm subocclusal) of pulp horn two in a first molar in the left lower jaw in a 3-years-old animal (dental age of 2 years and 4 months). A layer of odontoblasts (O) is visible in the periphery of the pulp. Due to shrinkage artifacts, the columnar morphology of the cells is not displayed very well. The predentin is clearly visible (light blue) (PD). At the border between the predentin and the mineralized dentin, globular structures are often visible indicating a mixture of linear and globular mineralization. More centrally, at the level of the pulp, blood vessels can be identified (Toluidin, 10x magnification). **(B)** A transverse decalcified section through root I of the left maxillary first molar of a 3-years old alpaca (dental age of 2 years and 4 months). A large free pulp stone (350 μm; yellow asterix) and multiple smaller attached pulp stones (red asterix; 20–50 μm), incorporated in the dentin can be perceived. The pulp, and its lining odontoblasts, have lost connection with the predentin due to a shrinkage artifact. Note the wider cemental thickness at the distal side (>300 μm) of this root I, compared to the mesial side (between 100 and 200μm). Also, a larger number and diameter of blood vessels can be found at the mesial side of this root compared to other sides (green asterix). The yellow box provides an enlargement of the large free present pulp stone (350 μm; Toluidin, 2.5x magnification).

Identification of the pulp cavity on histological sections and μ-CT images was straight-forward (Figure 2A). The pulp tissue in the alpaca follows the typical stratigraphic morphology of the vertebrate dentition with a peripheral lining of odontoblasts and a loose soft connective tissue in the central aspect. In-between the thin collagen fibers, fibroblasts, blood vessels and nerves can be found. Other identifiable structures at the level of the pulp included “false” pulp stones (*syn*. Denticles) consisting of concentric layers of mineralized tissue without the typical internal tubular structure, usually formed around blood thrombi, necrotic cellular material or collagen fibers (Figure 2B) (11, 14).

These false pulp stones were found in 10/27 (37.0%) sections of the pulp, thereby being represented in 8/9 (88.9%) teeth (Table 1). A total of 39 pulp stones could be detected of which 10.3% (4/39) were free ranging in the pulp cavity, and 89.7% (35/39) were incorporated in the dentin. The size of the pulp stones varied from 20 to 700 μm, whereby most pulp stones ranged in size from 50 to 200 μm. None of the pulp stones was associated with neighboring leukocytic infiltration. No “true” pulp stones, in which an internal tubular structure exists, were encountered.

**Table 1:** Presence of false pulp stones, both free and attached, on a section level (subocclusal, mid-tooth, apical) in nine alpaca cheek teeth.

	**False pulp stones**		
	**Free**	**Attached**	**Summary (free + attached)**
Sub-occlusal	0/9 (0)	2/9 (3)	2/9 (3)
Mid-tooth	1/9 (1)	3/9 (13)	3/9 (14)
Apical	2/9 (3)	5/9 (19)	5/9 (22)
Total	3/27 (4)	10/27 (35)	10/27 (39)

The total number of detected pulp stones is displayed between brackets for each specific level.

### Dentin

**Figure 3:**
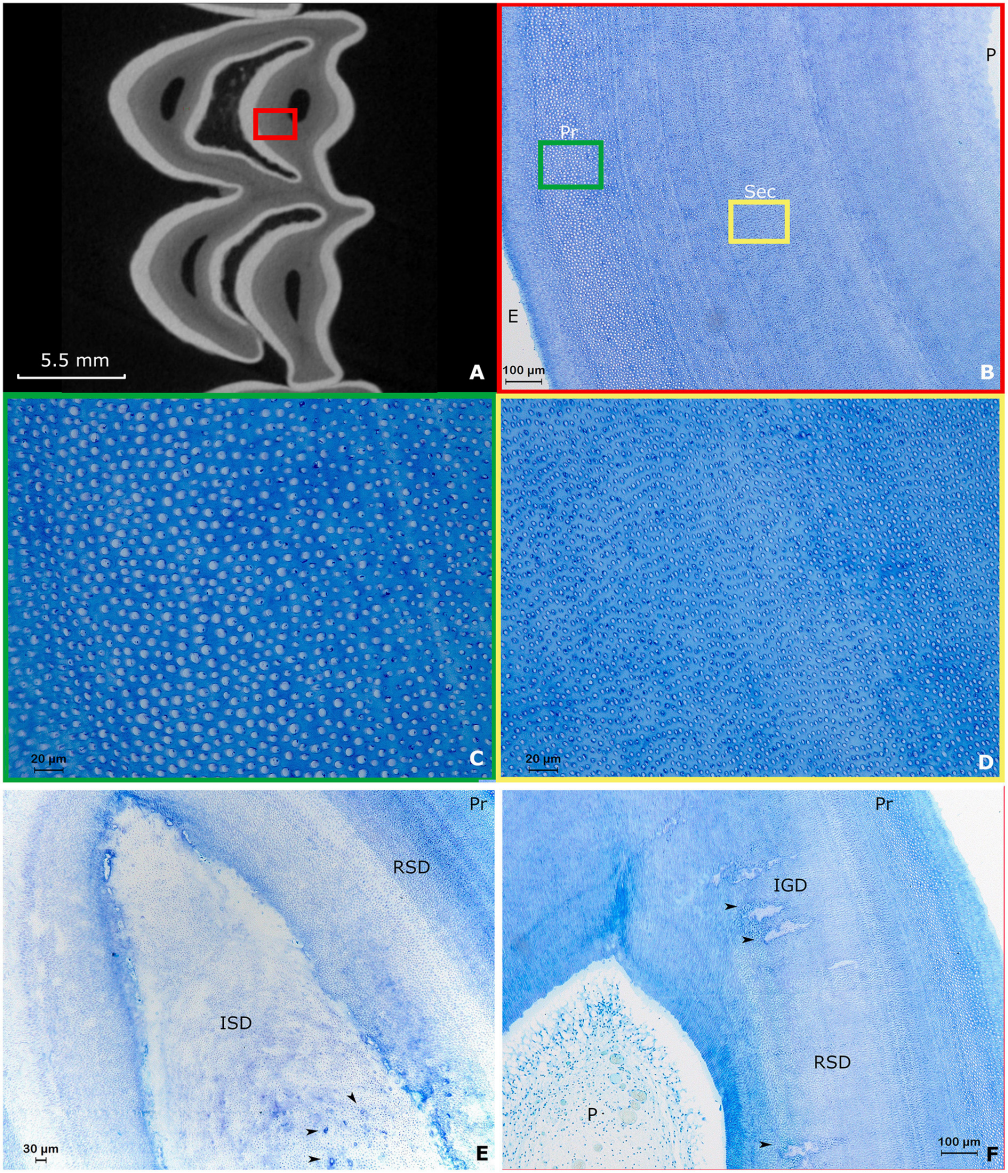
Typical arrangement of dentin in alpaca cheek teeth. Images **(A–D)**: Second molar in the right upper jaw of a 4 years and 3 months old alpaca (Dental age of 2 years and 6 months). **(A)** Transverse subocclusal 2D-image acquired using μ-CT. The mesial column of the tooth is placed up. Note the tooth is not yet fully developed, indicated by the ‘open infundibulum' at the level of the distal column. The enamel is displayed in white, the dentin in gray and the pulp horns are visible as black spots surrounded by dentin. A red square indicates the region studied using decalcified histology. **(B)** Overview of the normal histological arrangement of dentin in the alpaca. Close to the enamelodentinal junction, connecting dentin and enamel (E), primary dentin (Pr) can be found. When progressing toward the pulp (P), a transition occurs to secondary dentin (Sec). Note the multiple incremental lines present in the secondary dentin (Toluidin, 10x magnification). A green and yellow square indicate the enlarged region in **(C**,**D)**, respectively. **(C)** Detail of the primary dentin characterized by dentinal tubules surrounded by large amounts of peritubular dentin, whereby relatively small amounts of intertubular dentin are present (Toluidin, 40x magnification). **(D)** The secondary dentin is characterized by the absence of peritubular dentin. High amounts of intertubular dentin can be found in the secondary dentin. Remnants of odontoblast processes remain visible inside some dentinal tubules (Toluidin, 40x magnification). **(E)** Section through a first molar in the left lower jaw of a 3-years-old alpaca (Dental age 2 years and 4 months). Centrally, irregular secondary dentin (ISD) can be found above a pulp horn. Note the numerous vascular canals in this ISD indicated by black arrows. Regular secondary dentin (RSD) and primary dentin (Pr) can be found in the periphery (Toluidin, 10x magnification). **(F)** Section through pulp horn 4 of a second molar in the lower left jaw of a 5-years-old alpaca (Dental age 3 years and 3 months). Multiple hypermineralization zones are visible in the secondary dentin (black arrows). These zones can be defined as interglobular (IGD) dentin within the secondary dentin. This type of dentin is specifically associated with rapid deposition (11).

**Figure 4:**
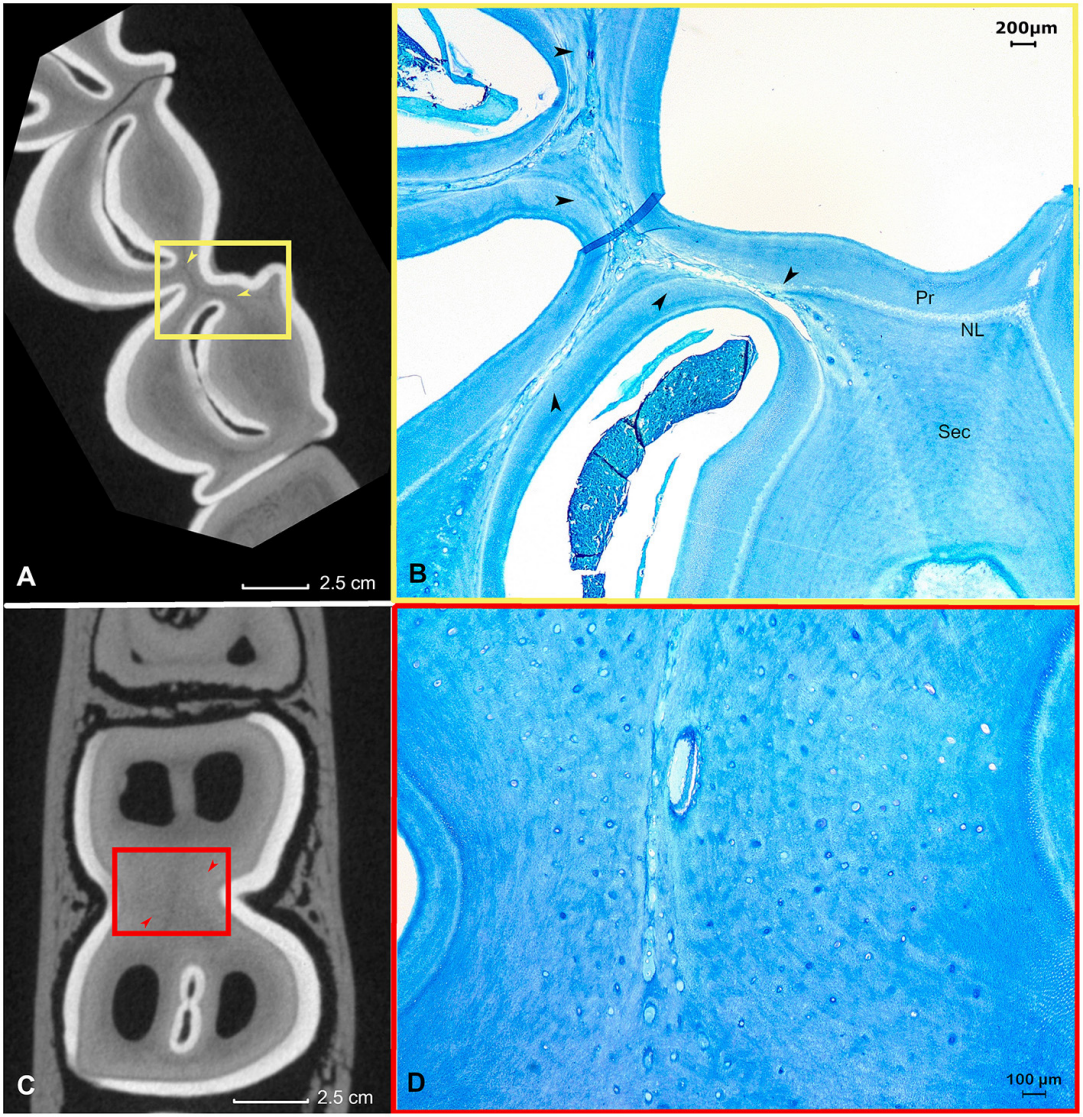
The histological appearance of vasodentin in a first molar in the left lower jaw in an alpaca of 3 years old (Dental age 2 years and 4 months). **(A)** μ-CT image of a sub-occlusal section at 4 mm depth. Note the dark gray lines indicating a “different type” of dentin (yellow arrows). A yellow box allows the orientation of the accompanying histological image **(B)**. **(B)** Histological appearance of the dentinal tissue at the level of the connection between the mesial and distal column (origin yellow box 4A). The dentin in this region is intermingled with remnants of blood vessels. This specific dental tissue was classified as vasodentin. Pr: primary dentin, NL: neonatal line, Sec: Secondary dentin (Toluidin, 2.5x magnification). **(C)** μ-CT image of a mid-tooth section at 10 mm depth. Note the dark gray region indicating a ‘different type' of dentin (red arrows). **(D)** Histological appearance of the magnified dentinal tissue at the center of the tooth. The dentin in this region is intermingled with remnants of blood vessels (Toluidin, 5x magnification).

**Figure 5:**
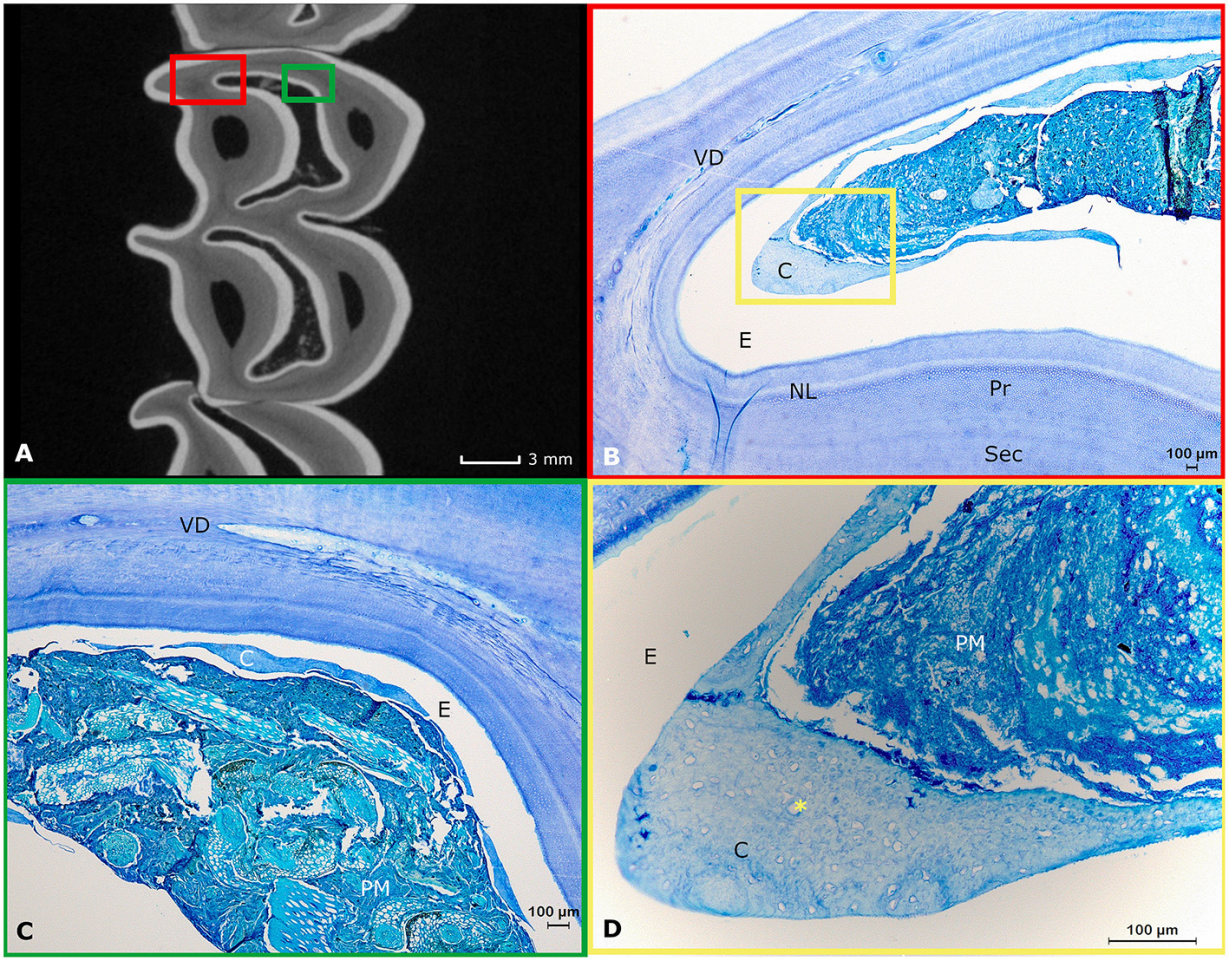
Histological appearance of infundibular structures. **(A)** A two-dimensional transverse μ-CT image of the subocclusal region (3 mm subocclusally) of a first molar in the left upper jaw originating from a 3-years-old alpaca shows the normal appearance of the infundibulae in alpaca cheek teeth (two centrally located white enamel rings; Dental age 2 years and 4 months). **(B)** Detail of the mesio-palatal edge of the mesial infundibulum (Histological presentation of content in red box Figure 4A). Infundibula in this species consist of wide enamel cusps largely filled with food, primarily consisting of plant material and little amounts of low cellular cementum (C). The narrow spaces created in-between the peripheral and infundibular enamel are prone to show the presence of vasodentin (VD). The normal structure of primary (Pr) and secondary dentin (Sec) is visible. A neonatal line (NL) is visible as a white line in this first maxillary molar. In the white space, enamel (E) used to be present prior to decalcification. **(C)** Histological representation of content indicated in the green box in **(A)**. In the center of this mesial infundibulum, even less cementum (C) can be perceived. The plant material (PM) is primarily occupying the former enamel cusp. Again, the white zone indicated by E was filled with enamel prior to any decalcification processes. Vasodentin is clearly represented in this specific histological section (VD). **(D)** Magnification of the zone indicated by a yellow box in **(B)**. A large amount of plant material (PM) and cementum (C). Detail of the infundibular cementum showing multiple cemental lacunae (^*^). The white zone indicated with an E was formerly filled with infundibular enamel prior to any decalcification processes.

Both maxillary and mandibular cheek teeth in the alpaca are characterized by large infundibula which minimize and shape the volume of the surrounding dentin (Figures 3A, 4, 5A). The structure of dentin displayed the typical arrangement of primary dentin in the periphery and secondary dentin in the more central zones (Figures 3B–F). The occlusal aspect of the pulp horn was consistently occluded by irregular secondary dentin. True tertiary dentin characterized by an irregular arrangement of incomplete dentinal tubules and a bulgy outline at the inner wall of the pulp cavity was observed in 15 out of 27 (55.6%) sections and 8 out of 9 (88.9%) included alpaca cheek teeth. Remarkably, a rather rare type of dentin previously characterized by the diffuse presence of ‘cavities', could be perceived in a subset of sections (Figure 4). Assuming its spongy appearance is created by remnants of rather large vessels infiltrating the dentin, the term ‘vasodentin', as previously described in other species, is used for further referencing (15). This vasodentin was found in 77.8% (21/27) of all sections, and 100% of all teeth. Prevalence on a subocclusal, mid tooth and apical level specifically, is displayed in Table 2. In the examined dental sections, vasodentin was perceived primarily in zones where pulp tissue had been present before during dental development. At a crown level, first and second molars are known to show an infolding of the peripheral enamel, thereby creating two separate columns. The area of the former communication between the mesial and distal column of the examined molar cheek teeth seemed specifically predisposed to hold large amounts of vasodentin. The specific structure of vasodentin could not be studied on the acquired μ-CT images. However, different shadings of gray can be perceived at locations where vasodentin is demonstrated histologically (Figure 4). The mode of mineralization showed to be a mixture of linear and globular calcification in all sections of all examined teeth (Figure 2A).

**Table 2:** Presence of vasodentin on a section level in nine alpaca cheek teeth.

	**Vasodentin**	
	**# pos sections**	**% pos sections**
Sub-occlusal	9/9	100%
Mid-tooth	7/9	77.8%
Apical	5/9	55.6%
Total	21/27	77.8%

A neonatal line, previously described as an incremental growth line in other species was found in 22.2% (2/9) of teeth, both at a first molar level and originating from the same youngest animal (dental age 2 years and 4 months; Figures 4, 5B) (16, 17). No neonatal lines were found in sections from the apical region. Additionally, in dentinal areas circumventing specific pulp horns, comparable incremental lines were identified in all included sections, indicating an incremental increase and a steady apposition of newly formed dentin (Figures 2B, 3B,E,F, 4B, 5B).

**Figure 6:**
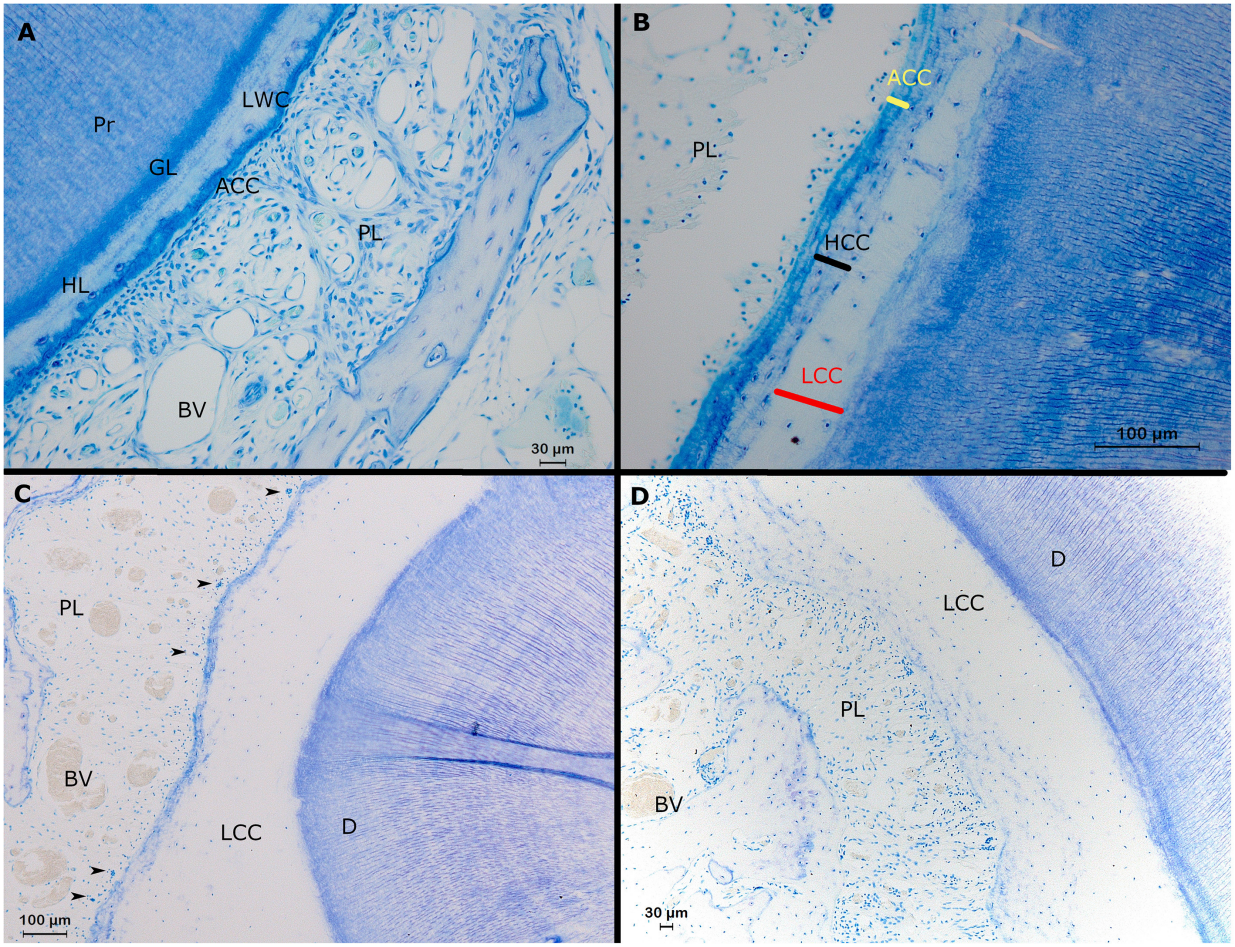
Histological representation of perceivable structures at the root level in alpaca cheek teeth. **(A)** Transverse decalcified histological section of a second molar in the right upper jaw (19 mm subocclusal) in a 4 years and 3 months old alpaca (dental age 2 years and 6 months). Adjacent to the primary dentin (Pr), the dentinocemental junction features a regular stratigraphic organization composed of a so-called granular layer of Tomes (GL) and a so-called hyalin layer of Hopewell Smith (HL). The dentinal tubules near the dentinocemental junction are organized in a parallel lining and show a typical branching. The dental cementum is composed of a layer of low cellular cementum (LWC) adjacent to the hyaline layer of Hopewell Smith. In this section, a small layer of acellular cementum (ACC) is visible in-between the low cellular cementum and the periodontal ligament (PL) with blood vessels (BV; Toluidin, 20x magnification). **(B)** Transverse section of a first molar in the left lower jaw in a 3-years-old animal (dental age 2 years and 4 months) showing the 3 different types of cementum at the level of alpaca cheek teeth. At the level of the dentinocemental junction, low cellular cementum can be found (LCC). Adjacent, more peripherally, a high cellular cementum (HCC) layer can be found. A layer of acellular cementum (ACC) forms a connection with the periodontal ligament (PL; Toluidin, 20x magnification). **(C,D)** Transverse sections originating from the same first premolar in the upper left jaw in a 3-years-old animal (dental age 2 years and 4 months; Toluidin, 10x magnification). **(C)** Buccodistal border of root I. A thick layer of low cellular cementum is present (LCC) adjacent to the dentin (D). Cell rests of Malassez are indicated by black arrows. Inside the periodontal ligament (PL), large blood vessels (BV) are visible. **(D)** Interradicular region of root 1 with only an extensive layer of low cellular cementum (LCC) connecting the dentin (D) and the periodontal ligament (PL). Some large blood vessels are visible within the periodontal ligament.

At the level of the apical sections, a well delineated stratigraphic arrangement of dentin was observed. As described in other mammalian species, a granular layer of Tomes and hyaline layer of Hopewell-Smith could also be differentiated in this species (Figure 6A).

**Figure 7:**
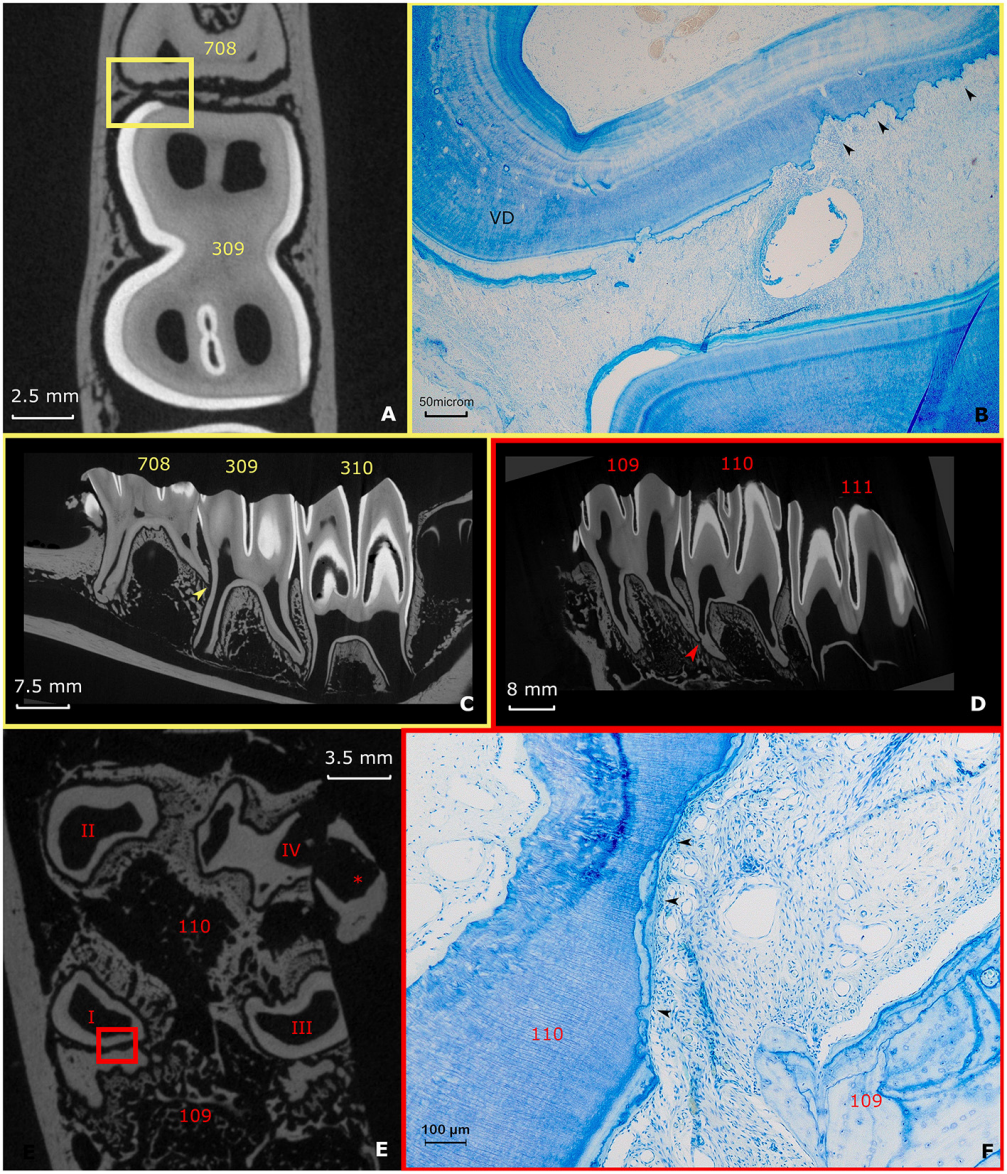
Root contacts and lytic zones **(A–C)** (yellow). A Root contact between the roots of a deciduous fourth premolar and permanent first molar in the left lower jaw in a 3-years-old animal. The apical end of root II of the deciduous fourth premolar (708) is in contact with the mid-tooth region of the first molar in the left lower jaw (309). **(A)** Transverse section 10 mm subocclusaly. Note the close proximity of the adjacent dental structures in this specific figure. Also, the lining of the dental tissue of the deciduous fourth premolar in the left lower jaw (708) appears irregular. A small yellow box indicates the range of the accompanying histological figure. **(B)** Histological decalcified section (10 mm subocclusal) of the region of contact indicated by a yellow box in **(A)**. Black arrows show the lytic front advancing in the dentin in the caudal region of root II of the deciduous fourth premolar in the left lower jaw. Note the presence of vasodentin (VD) at the root level of the deciduous fourth premolar in the left lower jaw (Toluidin Blue, 5x magnification). **(C)** Sagittal μ-CT image illustrating the root contact between the deciduous fourth premolar and first molar in the left lower jaw (yellow arrow) **(D–F)** (Red). Root contact between the roots of a first and second molar in the right upper jaw in a 4 years and 3 months alpaca. **(D)** Two-dimensional sagittal μ-CT section illustrating the root contact between the roots of a first and second molar in the right upper jaw (red arrow). **(E)** Transverse 17 mm subocclusal two-dimensional μ-CT image illustrating the region of contact between the roots of the first and second molar in the right upper jaw. Note the irregular border of the contacting root structures (red box). ^*^illustrates an artifact originating from the extraction of the dental arche from the skull. **(F)** Histological section at the apical region (17 mm subocclusal) of root I. Large amount of low cellular cementum present at a first molar in the right upper jaw (109). The mesial front of root I of a second molar in the right upper jaw (110) shows a lytic front proceeding into the dentin (black arrows). A small quantity of low cellular cementum is visible peripherally from the lytic front. (Toluidin, 10x magnification).

A specific finding in the examined teeth was the high frequency of physical contact between the roots of neighboring teeth. Root contacts were detected in 62.5% (10/16) of adjacent cheek teeth based on analysis of μ-CT images (Figures 7A,C–E). Histologically, several of these contacting roots showed massive resorptive lesions (Figures 7B,F) as observed in 8/10 (80%) mesial or distal zones of contacting roots.

These resorptive lesions extended through the cementum into the dentin and were characterized by the presence of necrotic tissue and clastic cells. Dentinal resorptive lesions were always covered by a very thin layer of cellular cementum providing attachment to the alveolar bone through the periodontal ligament (Figure 7F). Despite these obvious lytic zones present at the level of the apical dentin, no dental abnormalities were detected through macroscopical evaluation of the clinical crowns. The apical sections of teeth from 2 out of 9 animals (22.2%) also showed comparable resorptive lesions however without the presence of physical contact between adjacent roots as seen on μ-CT images. Again, these areas were always covered by proper cementum providing attachment to the periodontal ligament.

### Cementum

**Figure 8:**
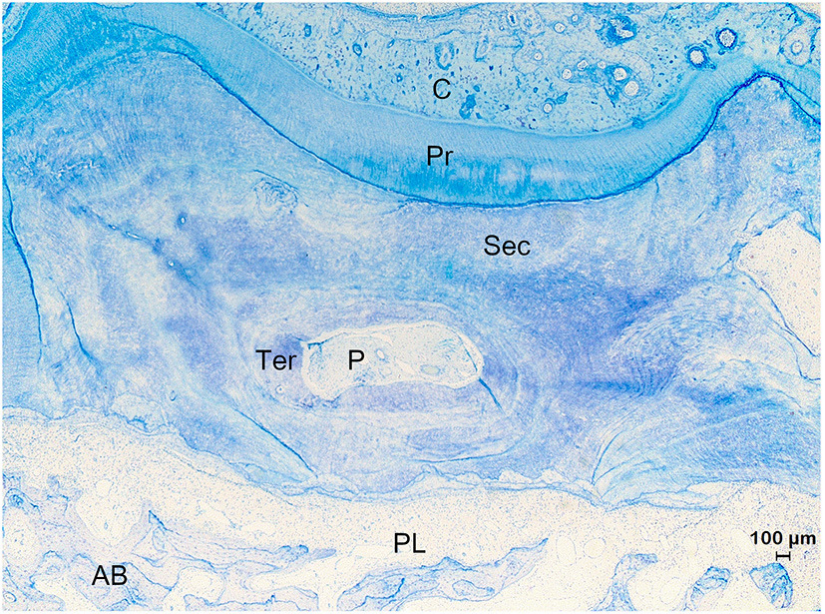
Histological section of the apical region of root IV of a 7 years and 5 months old first molar in the right upper jaw (Animal age of 8 years). Example of a tooth which experienced a drift. The top of the figure shows the tension side with cementum (C) holding vascular canals and irregular resting lines indicating a history of episodes of resorption and reparation. At the tension side, the tooth showed a movement in a direction away from the alveolar bone (AB) whereby large amounts of cementum were deposited to keep the alveolar space. At the pressure side (bottom of figure), the tooth was pressed against the alveolar bone causing resorptive processes to occur to remove hard substances and keep a periodontal ligament (PL). Different types of dentin including primary (Pr), secondary (Sec) and tertiary (Ter) are found. The root canal shows pulp tissue (P) (Toluidin, 2.5x magnification).

Only a relatively thin layer of cementum (most often up to 100-150 μm, occasionally around 900 μm) was present in the periphery of studied alpaca cheek teeth at a mid-tooth level, however the most apical root tips were characterized by thick layers of cementum (up to 2300 μm). The thickness of cementum layers was often variable at an apical level, though with a recognizable pattern over all sections. The cementum at the side of a root opposing another root of the same tooth was consistently thicker compared to sides of the roots directing toward the outer border of the concerning tooth. The older the teeth, the thicker the cementum layer at an apical level. The cementum structure is comparable as already described in all other vertebrates. The four discernable types of cementum in alpaca cheek teeth are displayed in Table 3. The differentiation of four different types of cementum was based on the presence of cementoblasts/cementoblast lacunae and the content of cementoblast lacunae, if present. Alpaca cheek teeth consistently have a thin (up to 70 μm) layer of so-called acellular extrinsic fiber cementum. This was primarily found at mid-tooth level as a delicate thin layer in contact with the periodontal ligament, which is consistent with findings in human cementum (18). Low cellular cementum and high cellular cementum are regarded as regular cementum with continuous and straight incremental lines (Figures 6B–D). Both types of cementum were found for all included cheek teeth. The fourth type of cementum, featuring an irregular distribution of cementoblast lacunae along with irregularly arranged incremental lines was referred to as irregular/reparative cementum. This type of cementum was only found in the apical sections of the 4 oldest included elements and in the apical section of a 4 years and 3 months old animal (dental age 2 years 6 months; Figure 8).

**Table 3:** Differentiation of the different types of cementum observed in alpaca cheek teeth based on the presence, concentration, and distribution of cementoblasts and/or cementoblast lacunae (CL).

**Types of cementum**	**Presence of cementoblasts/CL**	**# CL/10.000 μm^2^**	**CL Distribution**
Acellular extrinsic fiber cementum	No	NA	NA
Low cellular cementum	Yes	<6	Homogenous
High cellular cementum	Yes	>10	Homogenous
Irregular/reparative cementum	Yes	Not defined	Irregular

Both maxillary and mandibular cheek teeth showed the presence of an infundibulum composed of an outer enamel ring, internally aligned by a thin (30–200 μm) layer of cementum. The enamel did not show any interruptions or peculiarities on μ-CT (Figure 5A). The infundibulum appears to be filled largely with a heterogenous matter most likely consisting of food particles (Figures 5B–D). During histological processing, most of the infundibular content got lost due to a loss of attachment caused by decalcification of the surrounding enamel. In the remaining 3/27 histological sections, infundibular and peripheral cementum showed many similarities. These three histological sections showed a homogenous distribution of cementum lacunae which was categorized as low cellular cementum. No specific cellular regions nor blood vessels were encountered in this infundibular cementum (Figure 5D). Notable is the wide occlusal lingual/palatal to buccal width of the infundibula at the occlusal level, remarkably more pronounced in maxillary cheek teeth (Figure 5A). These large cups allow the accumulation of relatively large amounts of food particles in the center of the molars. However, no consequences of any inflammatory nature have been noted in surrounding dental tissues (Figures 5A–D).

### Periodontal ligament

The periodontal ligament of alpaca cheek teeth showed an arrangement of densely packed collagen fiber bundles intermingled with fibroblasts. Numerous and large (diameter up to 350 μm) blood vessels were found within the periodontal space, which can provide a cushioning action against forces of mastication. Remnants of Hertwig's epithelial root sheath, also termed cell rests of Malassez, were found at regular intervals in the apical sections, ranging from less than 1–4 islands per 100.000 μm^2^, and even higher frequencies in some mid tooth sections. Cell rests of Malassez were only present in the proximal fifth of the periodontal ligament, closest to the tooth. No species-specific properties of the periodontal ligament in the alpaca could be found.

## Discussion

Even though dental disease in alpacas is recognized as a severe health concern, the pathogenesis remains poorly understood (3, 19). Relevant dental pathology at the level of the incisors is found with a relatively low prevalence compared to similar abnormalities at the level of the cheek teeth in alpacas. Hence, apical infections, previously termed tooth root abscesses, are the most common dental condition affecting alpacas, primarily involving the cheek teeth (1). Recently, micro-computed tomographic studies have confirmed that adjacent tooth roots from different cheek teeth can physically contact each other (7, 8). However, up until now, no histological studies have been published focusing on the normal histological findings at the level of the cheek teeth in this species. Historically, data obtained from primarily equid histological studies have been used as material for extrapolation and as a basis for diagnosis and treatment (12, 20). The present study provides new insights into the normal histological arrangement and characteristics of specific dental tissues in alpaca cheek teeth.

Most histological findings for specific dental tissues in alpacas are in line with previously published material in other hypsodont and even brachydont species (11–14, 18, 21, 22). Nevertheless, the presence of vasodentin, interglobular dentin and a mixture of globular and linear mineralization suggest that development and mineralization of the cheek teeth in alpacas is a rather fast process. This process might occur so fast that the blood vessels at the level of the pulp might not have the time to withdraw because dentin formation occurs so quickly. This phenomenon could explain the diffusely dispersed vasodentin, most often found in areas which held the former pulp cavity. The vasodentin appears to be based on remnants of the pulp cavity in which there used to be a lot of blood vessels in former times. This is true for the very small areas, narrow channel-like structures, bridging larger areas of vasodentin. Despite vasodentin being only very seldomly described in other animals, in alpacas it seems to be a normal structure. Also, the high prevalence of interglobular dentin further supports the theory of rapid dentin formation and mineralization. This type of dentin is typically found as unmineralized or hypomineralized zones within the dentin (14). In humans, the presence of this interglobular dentin is also linked to a deficiency in vitamin D or high fluoride levels during dentin development (14). Furthermore, also the mixture of globular and linear mineralization in all investigated samples indicates a fast dentin deposition as also perceived in humans (14). All these findings appear correlated and underlined by the fact that the dentition appears in a very small range of time (23).

Vasodentin has been reported in a minority of species including sloths, armadillos, cats, and teleost fish as a softer and more porous variety of dentin (15, 24–26). The ample occurrence of vasodentin in the alpaca in comparison to other species, suggests the biomechanical strength of alpaca cheek teeth might be less, compared to cheek teeth in species where only regular orthodentin is found. These areas comprising large amounts of vasodentin can possibly be more susceptible to the development of fissures, fractures or even infectious disease processes. Overall, the low prevalence of fractures as reported in a large-scale prevalence study of dental disorders in this species, contradicts this hypothesis (3). As no structurally intact blood vessels nor soft connective tissue arrangements were identified within the vascular channels, it is assumed that the observed channels within the vasodentin are remnants of a vascular network which was vital during the development of this type of dentin. Further research is warranted to provide insights into the role of vasodentin in dental pathology of alpacas.

In wild animals, age determination is often performed based on the assessment of ‘dentin growth layers' or incremental lines found in the dentin of teeth. In addition, other extrinsic or intrinsic events may be recorded within the dentin. For example, in cetaceans (whales, dolphins, …), incremental lines can be found linked to birth (neonatal line), sexual maturity, weaning, lactation and environmental events (27). Also in human dentistry, incremental lines have been linked to illness and inadequate nutrition (14). Usually, this feature is less pronounced in animals in captivity due to the continuous availability and uptake of high-quality nutrition. Nevertheless, the extremely high prevalence of incremental lines in our study population, solely consisting of domesticated animals, might indicate that the nutritional status and/or other factors led to suboptimal conditions for continued dentin production from time to time. However, the understanding of the temporal framework of the development of incremental lines in the dentin of the alpaca is of primordial importance to draw any definite conclusions.

A specific incremental line, the neonatal line, is caused by the different physiologic changes at birth in humans and related to diet and behavioral changes during the first months of life in the dolphin (16, 17). This structure was found in 2 studied first molars, originating from one 3-years-old alpaca. This animal was the youngest animal included in the study population. These lines might also occur as a result of the fast dentin production in this species. Several incremental lines are often found in these teeth in alpacas rendering the identification of a distinct neonatal line at the level of the dentin difficult. Further studies in alpacas are necessary to allow any conclusion regarding the occurrence of neonatal lines and its possible association with time of formation and influencing factors which might provide usable information regarding development and mineralization times of first molars in the alpaca.

As in equids, a relatively high prevalence of pulp stones was found in the sections of our macroscopically normal teeth in the studied alpacas compared to reported numbers in humans (11). Eight out of 9 (88.9%) examined teeth showed at least one pulp stone. Pulp stones found in our study could be categorized as “false pulp stones” since they consisted of concentric layers of mineralized tissue without the typical dentinal internal tubular structure. These pulp stones are known to be formed predominantly around blood thrombi, necrotic cellular material or collagen fibers (14). Currently, no clarification exists on whether these pulpar calcifications should be considered pathological related to various external stimuli or just a natural phenomenon (28). The cause of pulpal calcification is largely unknown (28). In humans, a higher prevalence is associated with increasing age and local irritation, primarily consisting of attrition, abrasion, erosion, caries, periodontitis, dental restorations, orthodontic tooth movement and dental injury (29). Also, studies in humans and rats have shown excessive doses of vitamin D supplementation to be a causative factor for the formation of pulp stones (30, 31). The high prevalence of pulp stones is considered significant since their existence reduces the available surface and consequently the number of cells and vascularization of the pulp resulting in a reduced ability of dentin production (14). Nevertheless, no reports of increased vulnerability to tooth infection associated with the presence of pulp stones has been reported yet. However, pulp stones can interfere with endodontic therapies, including root canal debridement and enlargement (14). Therefore, the high prevalence of pulp stones should also be taken into consideration during the development of new treatments for dental pathology in alpacas.

A question remains whether the high prevalence of apical root resorption is a predisposition for secondary tooth pathology. Apical root resorption is frequently reported in human dentistry as a side effect caused by the traction and compressive forces initiated through treatment induced orthodontic movements (32). To our knowledge, similar findings have not been reported in a normal situation in any species. Nevertheless, the high prevalence of these root contacts in the studied specimens suggests this phenomenon to be present rather frequently in alpacas. A genetic component as a result of breeding cannot be excluded in this stage. No records are available on comparable research in wild animals. At histology, lytic zones were present in areas of root contacts between adjacent teeth. These were most likely induced by pressure, whereby large amounts of clastic cells are recruited, initiating the formation of lytic zones. The stress per unit surface area is expected to be relatively high causing resorption to extend through the cementum into the dentin. These areas in the cheek teeth of alpacas might indicate a lot of movement within the periodontal space of the tooth causing times of resorptive lesions followed by times of reparative processes. Another assumption holds that the described contact of the roots of neighboring teeth might imply the existence of a wider range of movement on the root level than normal. This might be an indirect effect of an abnormal/minimized wear of teeth. The clinical crowns remain longer than normal or are worn in an abnormal shape. In this case, the prolonged aspects of the tooth might act as a lever when masticatory forces are applied causing the roots to move in a wider range then normal thereby increasing the periradicular forces. In cats, which are fed vitamin D rich diets given their inability to produce vitamin D, an increased vitamin D activity has previously been associated with the presence of odontoclastic resorptive lesions and hypercementosis in one study (25). Also, in humans systemic disturbances have been described as causative factor (31, 33). However, later studies could not support this hypothesis since raised circulating levels of 25-hydroxyvitamin D could not be identified in both cats and humans affected by tooth resorption (34–36). Nevertheless, the involvement of an active vitamin D signaling in the pathology of tooth resorption in cats has been demonstrated (35). Prophylactic Vitamin D3 treatments are common practice in alpacas in large parts of the world characterized by prolonged periods of reduced UV-radiation (37, 38). Future studies investigating a possible link between vitamin D blood levels and the presence of apical root lysis might be of interest in this specific species. However, it is importance should not be overestimated and other factors including, but not limited to, phosphate, calcium and alkaline phosphatase levels might also play a role (39). By now we cannot conclude whether these resorptive areas will cause any pain to the animal as doubts regarding this subject also remain in horses affected by equine odontoclastic tooth resorption and hypercementosis (40). We do not know if therapeutical intervention is warranted at this stage (40–42).

It is not clear if the observation of root contacts in our study as well as its effect on tooth resorption could predispose to secondary lesions. Interestingly, apical infections are commonly encountered in alpaca cheek teeth (1, 43). The inflammatory nature of the resorptive process might act as predisposing factor to the development of apical infection in case bacterial inoculation of these tissues occurs through anachoresis caused by an increased blood flow, or periodontal disease. The spread of infections over different cheek teeth might also be facilitated by the present root contacts in this species. The authors have seen many clinical cases of diffuse osteomyelitis (with or without apical disease) along dental arches (unpublished data). On the contrary, in horses, apical infection often remains confined to one specific element (44). Future studies are necessary to allow an elucidation of this possible association.

## Conclusion

The histological characteristics of dental tissues at the level of the cheek teeth in alpacas are similar to findings in other hypsodont and brachydont species. Dentin formation and mineralization is expected to be a very quick process in the alpaca characterized by rapidly formed softer and more porous vasodentin and hypermineralized areas incorporated in the dentin. An interesting finding was the presence of lytic regions in the dentin in all apical sections. The obtained results lead to novel insights into the pathogenesis of dental disease in the alpaca. Nevertheless, further histopathological studies of diseased teeth remain necessary to allow a better understanding of the etiopathogenesis of dental disorders in this species.

## Data availability statement

The original contributions presented in the study are included in the article/supplementary material, further inquiries can be directed to the corresponding author.

## Ethics statement

Ethical review and approval was not required for the animal study because the used specimens were obtained from cadavers. Cause of death was unrelated to this study. Written informed consent for participation was not obtained from the owners because a general written consent for educational and scientific use was obtained.

## Author contributions

KP designed the study, collected the specimens, analyzed the acquired images, and wrote the manuscript. CS contributed to the study design, histological processing of the samples, analysis of the acquired images, and revision of the manuscript. MB contributed to the acquirement of micro-computed tomographic studies and revision of the manuscript. JV contributed to the histological processing of the samples. IJ contributed to the acquirement of micro-computed tomographic studies. LV contributed to the study design and revision of the manuscript. KC contributed to the study design, analysis of the acquired images, and revision of the manuscript. All authors contributed to the article and approved the submitted version.

## Funding

The Ghent University Special Research Fund (BOF-UGent) is acknowledged for the financial support to the Centre of Expertise UGCT (BOF.EXP.2017.0007).

## Conflict of interest

The authors declare that the research was conducted in the absence of any commercial or financial relationships that could be construed as a potential conflict of interest.

## Publisher's note

All claims expressed in this article are solely those of the authors and do not necessarily represent those of their affiliated organizations, or those of the publisher, the editors and the reviewers. Any product that may be evaluated in this article, or claim that may be made by its manufacturer, is not guaranteed or endorsed by the publisher.
